# Disturbances in Maternal Steroidogenesis and Appearance of Intrauterine Growth Retardation at High-Altitude Environments Are Established from Early Pregnancy. Effects of Treatment with Antioxidant Vitamins

**DOI:** 10.1371/journal.pone.0140902

**Published:** 2015-11-11

**Authors:** Victor H. Parraguez, Sandra Mamani, Eileen Cofré, Giorgio Castellaro, Bessie Urquieta, Mónica De los Reyes, Susana Astiz, Antonio Gonzalez-Bulnes

**Affiliations:** 1 Faculty of Veterinary Sciences, University of Chile, Casilla 2, Correo 15, La Granja, Santiago, Chile; 2 Faculty of Agricultural Sciences, University of Chile, Casilla 2, Correo 15, La Granja, Santiago, Chile; 3 International Centre for Andean Studies, University of Chile, Casilla 2, Correo 15, La Granja, Santiago, Chile; 4 Comparative Physiology Lab, SGIT-INIA, Av. Puerta de Hierro s/n, 28040, Madrid, Spain; 5 Department of Veterinary Medicine, University of Sassari, Via Vienna 2, 07100, Sassari, Italy; University of Southampton, UNITED KINGDOM

## Abstract

Pregnancies at high-altitudes are influenced by hypoxia and oxidative stress and frequently affected by IUGR. However, a common thought is that early pregnant women visiting altitude have no major complications for gestation development, since IUGR is developed during the second half of pregnancy. Thus, using a well-characterized sheep-model, we aimed to determine whether long- and/or short-term exposure to high-altitude may affect maternal steroidogenesis and therefore embryo-fetal growth from conception. The second aim was to differentiate the relative role of hypoxia and oxidative stress by assessing the effects of supplementation with antioxidant agents during this early-pregnancy stage, which were previously found to be useful to prevent IUGR. The results indicate that both long- and short-term exposure to high-altitude causes disturbances in maternal ovarian steroidogenesis and negatively affects embryo-fetal growth already during the very early stages of gestation, with the consequences being even worsened in newcomers to high-altitude. The supply of antioxidant during this period only showed discrete effects for preventing IUGR. In conclusion, the present study gives a warning for clinicians about the risks for early-pregnant women when visiting high-altitude regions and suggests the need for further studies on the effects of the length of exposure and on the interaction of the exposure with the pregnancy stage.

## Introduction

Pregnancies developed at high-altitude are characterized by maternal and therefore fetal hypoxia, leading to an increased risk of intrauterine growth retardation (IUGR; [1]) with small-for-gestational-age newborns and increased infant mortality [2–6]. Occurrence of IUGR by high-altitude, independently of other concomitant social and economic factors [2, 7, 8] is estimated at around 17%, whilst IUGR at low-altitude is estimated at 6.0% [3]. Hence, the study of the deleterious effects of altitude on pregnancy is essential. Firstly, because around 140 million people live at altitudes above 2500 m.a.s.l.; second, because another 40 million people visits these regions yearly [9]. Therefore, many women in all stages of pregnancy visit highlands every year. However, in spite of the data indicating aggravated effects in newcomers to high-altitude [5, 10–12], the common idea is that visiting altitude causes no major complications for healthy early-pregnant women since IUGR is developed during the second half of pregnancy (e.g.: the Consensus Statement of the UIAA Medical Commission, which can be found freely available at http://www.theuiaa.org/upload_area/files/1/UIAA_MedCom_Rec_No_12_Women_at_Altitude_2008_V1-2.pdf)

The truth is that the safety of visiting high-altitude regions during early pregnancy has not been thoroughly studied. We can hypothesize that the assumption of a safe stay of early-pregnant women at altitude may be related to the fact that, in the developed world, 60% of IUGR offspring is identified as caused by placental insufficiency due to inadequate development [13, 14]. Deficiencies in placental development lead to progressive disturbance of oxygen and nutrient exchange between mother and fetus, with the consequences becoming apparent during the second half of pregnancy [15]. However, recent studies suggest that intrauterine growth trajectory is programmed from the initial stages of gestation [16–19].

On the other hand, we can hypothesize that the effects of foetal hypoxia due to placental deficiencies may be different to the effects of fetal hypoxia due to maternal hypoxia, which may be present at the very early stages of pregnancy, prior to the development of the placenta. The beginning of pregnancy, in the pre- and early-implantational stages, is characterized by hypoxic conditions [20–22]; however, there are no data on the effects of superimposed maternal hypoxia. Moreover, when exposure to altitude is not only related with hypobaric hypoxia but also with increased oxidative stress, due to incomplete oxygen reduction; oxidative stress may negatively affect embryo and fetal development [23].

Having in mind the ethical issues limiting research in human beings, we have conducted several studies on the effects of hypobaric hypoxia at high-altitude environment on pregnancy outcomes using a well-recognized animal model of pregnancy: the sheep [24–27]. The main purpose of the study is both, biomedical and in animal husbandry (about 25 million people living in the Andean and Qinghai-Tibetan plateaus depend economically on sheep breeding; [28]). Studies in our sheep model have shown similar results to human data, with the effects of altitude being most deleterious in human and sheep newcomers to altitude, with remarkable changes in placental weight, size and vascularization [29] as well as sharply decreased fetal growth and reduced birth-weight [29–31]. In mammals, the processes of placental neovascularization and angiogenesis are strongly determined by the secretion of steroid hormones [32]. In sheep, pregnancy is fully supported by placental steroidogenesis from Days 50–60 onwards [33]. We have found, in females exposed to high-altitude, that steroid secretion is disturbed during the last two-thirds of pregnancy (from day 70 of pregnancy to delivery), which negatively influences the fetal development [31]. Ovarian steroid secretion is also disturbed at high-altitude [34]; specifically, the progesterone secretion is diminished during the first days of pregnancy (i.e. during the luteal phase prior to implantation [35]). However, there are no specific studies on maternal steroidogenesis during the early gestation, during the first third of pregnancy. Thus, the first objective of this study was to determine whether long- and/or short-term exposure to high-altitude negatively affects secretion of progesterone and 17b-oestradiol and/or embryo-fetal growth at early stages of pregnancy (from Day 1 to 60).

In previous studies, we have found that the deleterious effects of oxidative status induced by high altitude may be prevented by daily administration of the antioxidant vitamins C and E [31]. The treatment enhances placental steroidogenesis and favors placental function and fetal development at the last two-thirds of pregnancy; increasing therefore newborns weight and viability. Hence, the second aim of the present study was to differentiate the relative role of hypoxia and oxidative stress at early pregnancy by assessing the effects of supplementation with antioxidant agents on steroidogenesis and and/or embryo-fetal growth.

## Materials and Methods

### Ethics statement

The experimental study was designed and performed in agreement with the International Guiding Principles for Biomedical Research Involving Animals (Council for International Organization of Medical Sciences, World Health Organization) and was approved by the Bioethics Review Committee of the Faculty of Veterinary and Animal Sciences, University of Chile (which is the named Institutional Animal Care and Use Committee, IACUC), as well as by the Bioethics Advisory Committee of the Chilean National Commission for Scientific and Technological Research (CONICYT, Chile).

### Animals and management

The experiment involved 36 Creole ewes (Chilean mix breed developed from Churra and Manchega Spanish breeds, BW = 42.2±2.3 Kg; BCS = 2.6±0.2) from the experimental flocks of the University of Chile, with a history of normal pregnancies and deliveries, which completed pregnancy after estrus synchronization and mating. Twelve of the 36 ewes were native to HA (group HH; descendants of sheep introduced to the Andean high plateau by Spanish settlers almost 500 years ago) and, at the beginning of the experiment, were allocated in a single pen at the animal facilities of the International Centre for Andean Studies (INCAS, University of Chile; ~ 3600 m.a.s.l., barometric pressure ~ 667 hPa). Other 24of the 36 ewes were native to low altitude (~500 m.a.s.l.; barometric pressure ~ 990 hPa) and were selected on the basis of similar phenotypes, body weights and age to the females in the group HH. Five days before the start the experimental protocol, half of these 24 animals native to low altitude were moved to the INCAS facilities to join the HH ewes (group LH, n = 12), whilst the other half were maintained at low altitude (group LL, n = 12). Animals were provided with alfalfa hay daily (2 kg/animal/day given in morning and evening similar rations, DM = 89.8%, ME = 10.6 MJ/kg, CP = 14.0%) and fresh water *ad libitum*. The food supply was calculated to satisfy the daily ovine requirements in gestation [36] and given individually to each sheep to assess possible refusals; there were no differences in food intake among groups that could be related with differences in reproductive outputs.

To determine the relative role of hypoxia and oxidative stress and the possible usefulness of antioxidant treatments, the 12 ewes in each group (HH, LH and LL) were randomly divided into two equal subgroups allocated in two different pens. One subgroup remained as control (maintaining the identification as groups HH, LL and LH, n = 6 each), whereas the other subgroup was were daily orally supplemented, early in the morning with 500 mg of vitamin C and 350 I.U. of vitamin E administered with 0.3 kg of alfalfa (groups HHV, LHV and LLV, n = 6 each). Once the supplemented alfalfa was fed, the morning ration was completed. Hence, six experimental groups were formed (n = 6 in each group). Four groups were kept at high altitudes: group HH (native to high-altitude without vitamins), group HHV (native to high-altitude with vitamins), group LH (native to low-altitude and taken to high-altitude without vitamins) and group LHV (native to low-altitude and taken to high-altitude with vitamins). Two additional groups of ewes native to low-altitude were maintained at low-altitude, with and without vitamin supplementation (groups LLV and LL, respectively).

Estrous cycles were synchronized from the day following the start of the antioxidant supply in the treated groups, by the administration of two i.m. doses of 125 μg of cloprostenol (Ovolute^®^, Drag Pharma, Santiago, Chile) given 9 days apart. Estrus detection was performed daily with trained vasectomized males introduced into the females' pens at the day after the second cloprostenol dose. The chests of the males were painted daily with a mixture of vegetable oil and colored powder, so any ewe in heat was detected on the following day by the observation of a colored rump. Five days after, vasectomized rams were changed by fertile males for mating on the following estrus.

### Sampling and measurements

Catheterization of the maternal blood vessels allows to obtain frequent blood samples without inducing stress by repeated venipuncture. Thus, on the same day of antioxidant supply, venous and arterial catheters (2.5 mm internal diameter, Tygon^®^, Saint Gobain Performance Plastics, Akron, Ohio, U.S.A.) were implanted in the corresponding left pedal vessels, under ketamine anesthesia (20 mg/kg i.m. of ketamine clorhidrate; Ketamil^®^, Troy Laboratories, Smithfield, Australia). Once implanted, the catheters were filled with heparinized saline (1000 IU/mL) to prevent clot formation. Catheters were then passed through the subcutaneous tissue to the left flank of the ewe where they were exteriorized and placed in a canvas pocket attached to the skin.

Arterial blood samples (1 mL) were taken for evaluation of partial pressure of oxygen (P_a_O_2_) and carbon dioxide (P_a_CO_2_), hemoglobin concentration (Hb), saturation of hemoglobin by oxygen (SatHb) and pH. These samples were obtained at the beginning, at the middle and at the end of sampling period (Days 12, 32 and 60 of pregnancy). Measurements were done in an IL Synthesis 25™ gas analyzer (Instrumentation Laboratory, Lexington, MA, U.S.A.), calibrated according to local atmospheric pressure and ovine temperature.

Venous blood samples (3 mL) were taken to assess the endocrine environment that sustains early pregnancy, through measurement of plasma progesterone and 17 β-estradiol concentrations. Samples were drawn in heparinized syringes every four days, starting at day 1 after mating, during a total period of 60 days (a total of 16 venous blood samples per ewe). Blood was centrifuged at 1200 g for 5 minutes and the obtained plasma was stored at -20°C until assayed. Estradiol 17 β was measured in duplicate by a solid phase radioimmunoassay (RIA), using 100 μL plasma aliquots and reagents and techniques provided by Diasource E2-RIA-CT kit^®^ (Diasource Immunoassays SA, Louvain-la-Neuve, Belgium). The assay sensitivity was 2 pg/mL (7.34 pM). The intra- and inter-assay coefficients of variation were 5.9 and 10.1%, respectively. Progesterone was analyzed in 100 μL plasma aliquots by a solid phase radioimmunoassay (RIA), using reagents and techniques provided by Coat-a-Count Progesterone^®^ (Siemens Healthcare Diagnostic Inc., Los Angeles, CA, USA). The samples were measured in duplicate, and the assay sensitivity was 0.031 ng/mL (0.1 nM). The intra- and inter-assay coefficients of variation were 4.0 and 5.3%, respectively.

Oxidative stress biomarkers were also measured in the same venous blood samples. Protein carbonyl groups concentrations were determined by a spectrophotometric method, following the protocol described by Reznick and Packer [37]. Plasma malondialdehyde concentrations were measured by HPLC with fluorescence detection using the thiobarbituric acid assay described by Lastard et al [38]. Total antioxidant capacity was assessed in plasma through the Total Radical-Trapping Antioxidant Parameter technique described by Wayner et al [39] and modified by Lissi et al [40].

Assessment of pregnancy was performed by two experienced sonographers (VHP and AGB) in a blinded fashion, using a real-time B-mode ultrasound equipment (Aloka SSD 500, Tokyo, Japan) fitted with linear transrectal probes (7.5 MHz until Day 40 of pregnancy and 5.0 MHz later on). From the Day 10 after mating, the ewes were examined every day until the observation of the embryonic heartbeat, and every 4 days from this moment to Day 60 of pregnancy to evaluate the embryo-foetal growth. Measurements were performed with the internal calipers of the ultrasound equipment and included head, thorax and abdominal diameters from the first Day of embryo visualization onwards, in order to acquire the growth-slope curves as previously described [28].

Finally, the individual bodyweight of each neonate was assessed at birth.

### Statistical analysis

Data on steroid hormones, arterial gases, biomarkers for oxidative stress, gestational age in which an embryo with heartbeat could be detected by ultrasound examination and birth weight of the lambs were compared by analysis of variance, using the general linear model procedure (GLM; SAS Institute Inc., Cary, NC, USA). Comparisons were made using two statistical models. The first model was used to test the effect of altitudinal status, including two cross factors: the place of birth or origin of the animals and the place where the pregnancies were studied. The second model took into consideration the previous two factors in addition to the antioxidant vitamin supplementation. Interactions among factors were also analyzed. When significant differences were found, Duncan’s test was used to determine the groups among which the differences were statistically significant. Areas under curve for plasma 17-β oestradiol and progesterone along the gestational period were also compared by means of analysis of variance. In addition, regression curves were obtained from embryo-fetal biometric variables as function of the gestational age. To establish eventual differences in embryo-fetal growth patterns, the slopes of the curves (previously linearized by logarithmic transformation) were compared using the method of comparison of regression lines in two levels of a categorical factor, calculated by StatGraphics Centurion XV software, Version 2.15.06 (StatPoint Technologies, Inc., Warrenton, VA, USA). Significant differences were considered when P≤ 0.05. The results are expressed as means ± SEM.

## Results

### Effects of altitude, altitudinal status and antioxidant treatment on maternal arterial blood gases and oxidative stress

The results of the assessment of arterial variables related to oxygen transport are shown in Table 1. There was no statistically significant difference in the values of arterial gases within the groups with time, when comparing the values at the beginning, middle and at the end of the sampling period; thus, average values for each arterial variable during the experimental period were calculated for each experimental group. In brief, the animals exposed to high-altitude (groups HH and LH) showed blood gas changes consistent with a state of hypoxemia by exposure to a hypobaric environment (low P_a_O_2_, P_a_CO2 and SatHb, with high Hb). The antioxidant vitamins improved the SatHb in the group LHV (P<0.05).

**Table 1:** Arterial partial pressure of oxygen (P_a_O_2_), carbon dioxide (P_a_CO_2_), hemoglobin concentration (Hb), percentage of hemoglobin saturated by oxygen (Sat Hb) and pH in sheep in early pregnancy: effects of long or short time exposure to altitude and of supplementation with vitamins C and E.

Group	P_a_O_2_ (mm Hg)	P_a_CO_2_ (mm Hg)	Hb (mg/dL)	Sat Hb (%)	pH
LL	96.4±2.6^a^	39.6±1.8^a^	10.0±1.8^b^	94.4±5.0^a^	7.44±0.01
LLV	97.3±1.3^a^	39.3±2.1^a^	9.9±0.9^b^	96.9±2.2^a^	7.42±0.01
LH	55.4±5.8^c^	30.9±4.1^b^	13.4±2.6^a^	76.1±7.3^c^	7.48±0.02
LHV	56.9±3.5^c^	32.4±2.4^b^	12.8±1.0^a^	80.4±3.3^b^	7.46±0.04
HH	61.0±4.7^b^	28.4±3.8^b^	12.5±1.1^a^	81.5±3.7^b^	7.51±0.03
HHV	62.5±5.5^b^	27.0±4.1^b^	12.5±0.7^a^	82.1±3.4^b^	7.52±0.02

Different superscript letters indicate significant differences among groups (P<0.05). LL: low altitude native sheep maintained at low altitude, without vitamins supplementation; LLV: low altitude native sheep maintained at low altitude, supplemented with vitamins; LH: low altitude native sheep taken to high altitude, without vitamins supplementation; LHV: low altitude native sheep taken to high altitude, supplemented with vitamins; HH: high altitude native sheep maintained at high altitude, without vitamins supplementation; HHV: high altitude native sheep maintained at high altitude, supplemented with vitamins.

**Fig 1:**
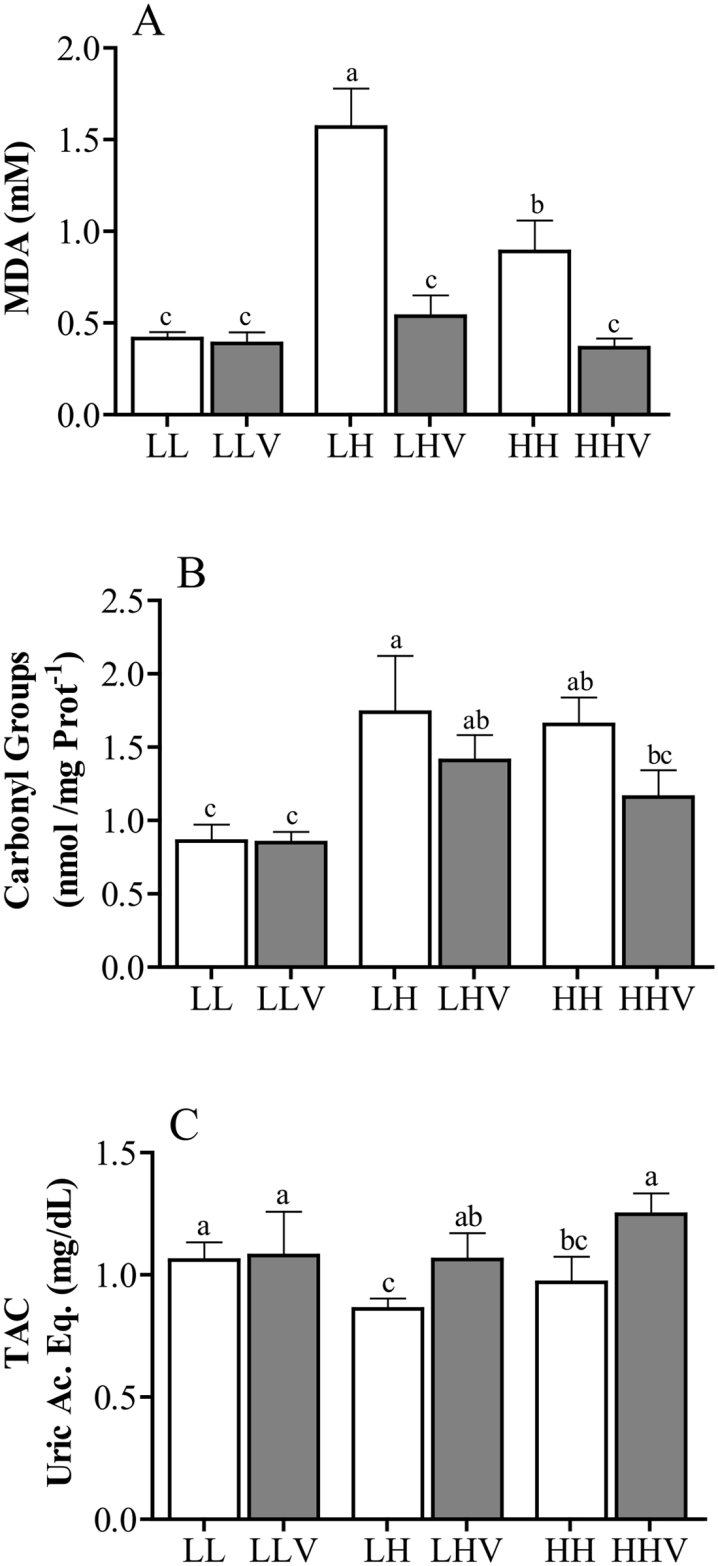
Oxidative stress biomarkers during the first 60 days of gestation in sheep pregnancies developed at high or low altitude. Mean (± SEM) values of plasma malondialdehyde (panel A), carbonyl groups (panel B), and total antioxidant capacity (panel C). HH: ewes native to highlands whose gestation took place at high altitude, without vitamins supplementation; HHV: ewes native to highlands whose gestation took place at high altitude, with vitamin supplementation; LH: ewes native to lowland whose gestation took place at high altitude, without vitamin supplementation; LHV: ewes native to lowland whose gestation took place at high altitude, with vitamin supplementation; LL: ewes native to low-altitude maintained at low-altitude, without vitamin supplementation; LLV: ewes native to low-altitude maintained at low-altitude with vitamin supplementation. The different letters above the bars indicate significant differences among groups (P<0.05).

The biomarkers for oxidative stress are shown in Fig 1. No statistically significant difference was found in the total antioxidant capacity among groups of different altitudinal status, but plasma concentrations of malondialdehyde and protein carbonyl groups were affected by the altitude. Plasma malondialdehyde concentrations were the highest in LH group (P<0.05), and higher in the HH group than in the LL group (P<0.05). The protein carbonyl groups were similar in HH and LH groups, and higher in both groups when compared to those of LL group (P<0.05). The administration of antioxidants improved the total antioxidant capacity in both HH and LH females (P<0.05) and reduced malondialdehyde concentrations in HH and LH females (P<0.05) to similar levels to those found in the LL group. Carbonyl levels were not significantly influenced by the antioxidants supply.

### Effects of altitude, altitudinal status and antioxidant treatment on stereidogenesis

**Fig 2:**
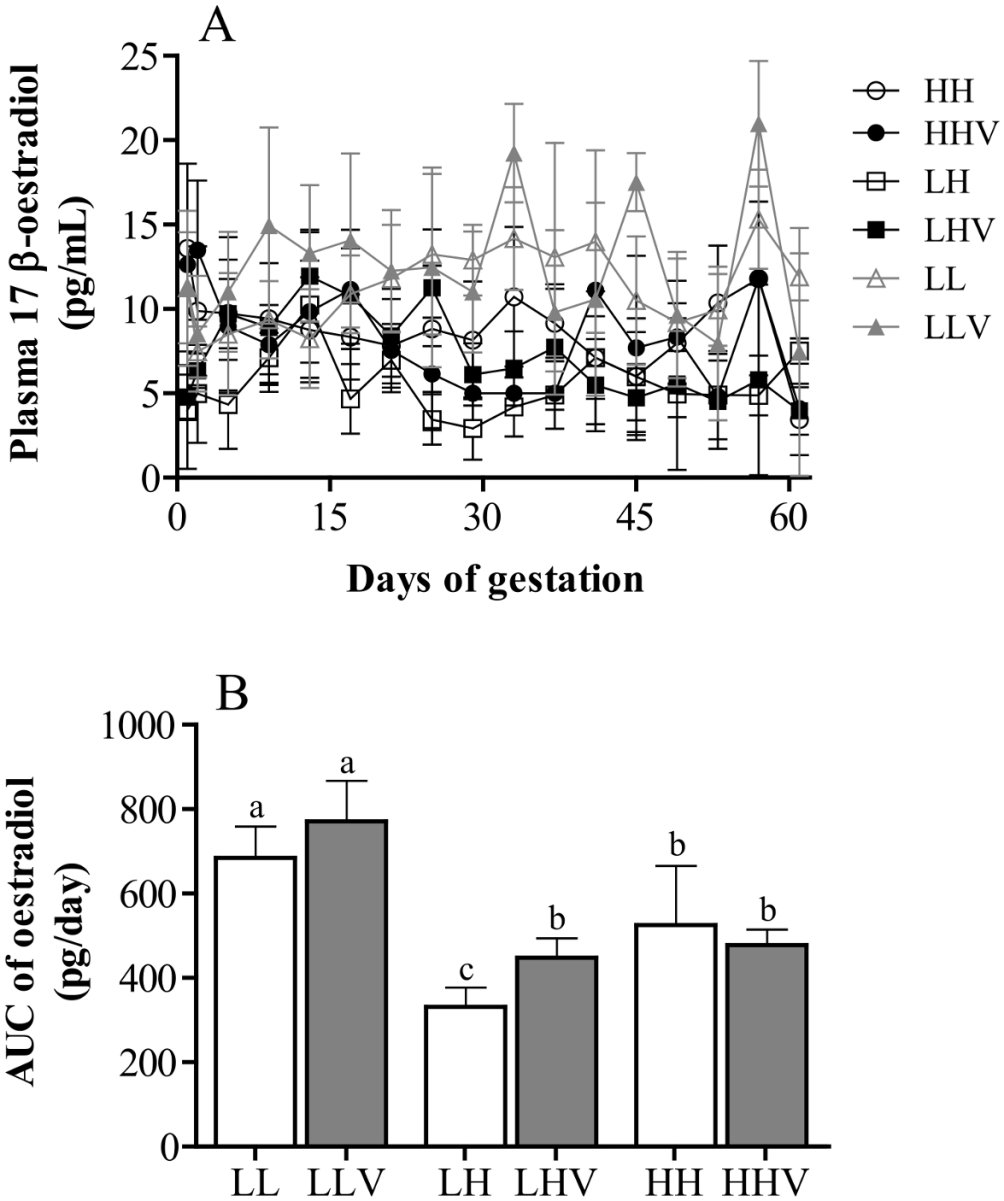
Daily profiles (panel A) and area under the curve (AUC, panel B) of plasma concentrations of 17β-oestradiol (mean ± SEM) during the first 60 days of gestation in sheep pregnancies developed at high or low altitude. HH: ewes native to highlands whose gestation took place at high altitude, without vitamins supplementation; HHV: ewes native to highlands whose gestation took place at high altitude, with vitamin supplementation; LH: ewes native to lowland whose gestation took place at high altitude, without vitamin supplementation; LHV: ewes native to lowland whose gestation took place at high altitude, with vitamin supplementation; LL: ewes native to low-altitude whose gestation took place at low-altitude, without vitamin supplementation; LLV: ewes native to low-altitude whose gestation took place at low-altitude with vitamin supplementation. In panel B, the different letters above the bars indicate significant differences among groups (P<0.05).

**Fig 3:**
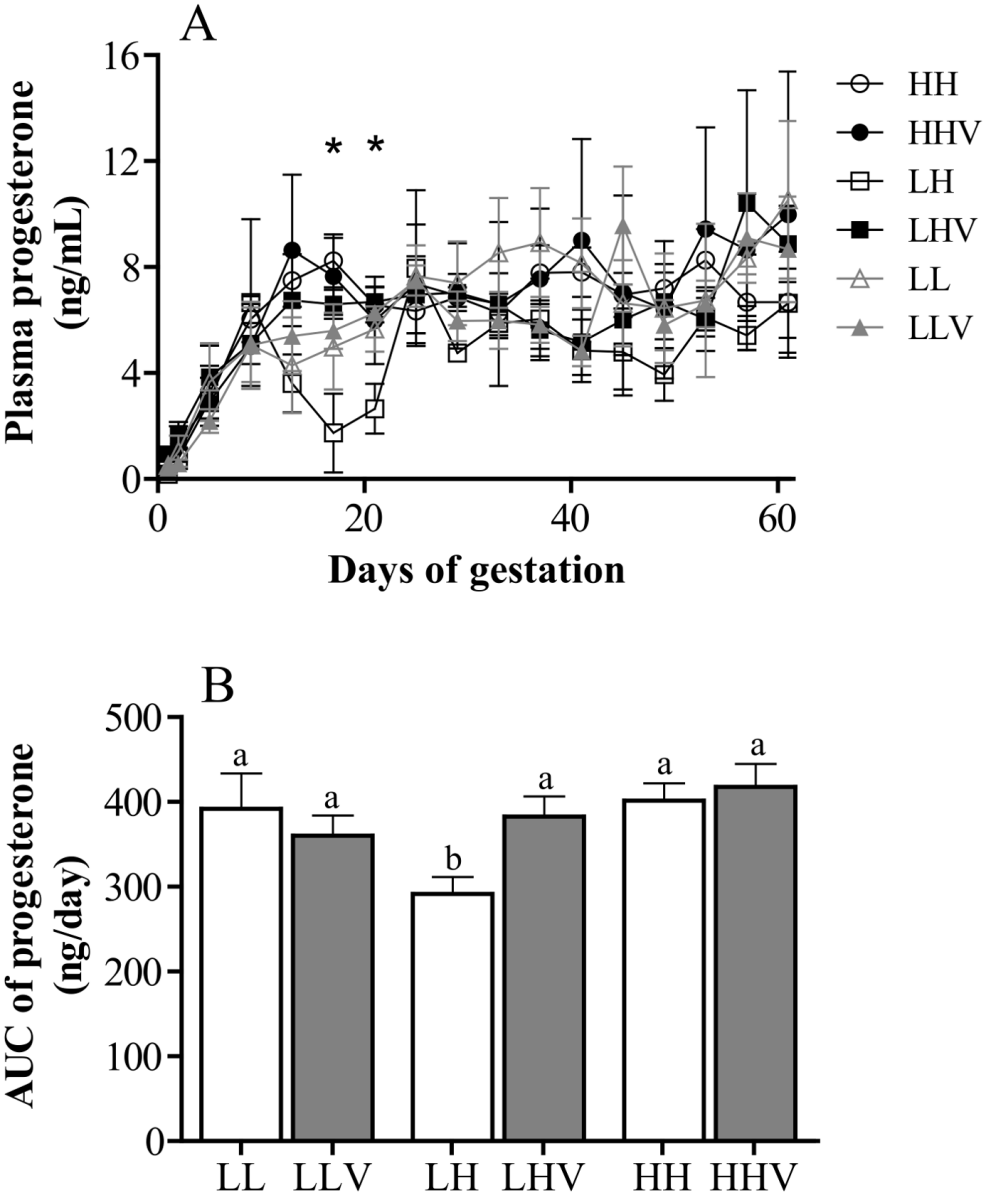
Daily profiles (panel A) and area under the curve (AUC, panel B) of plasma concentrations of progesterone (mean ± SEM) determined during the first 60 days of gestation in sheep pregnancies developed at high or low altitude. HH: ewes native to highlands whose gestation took place at high altitude, without vitamins supplementation; HHV: ewes native to highlands whose gestation took place at high altitude, with vitamin supplementation; LH: ewes native to lowland whose gestation took place at high altitude, without vitamin supplementation; LHV: ewes native to lowland whose gestation took place at high altitude, with vitamin supplementation; LL: ewes native to low-altitude whose gestation took place at low-altitude, without vitamin supplementation; LLV: ewes native to low-altitude whose gestation took place at low-altitude with vitamin supplementation. In panel A, asterisks above values of days 17 and 21, indicate that averages of LH group are different of all of the other values (P<0.05). In panel B, the different letters above the bars indicate significant differences among groups (P<0.05).

The results of the assessment of blood steroid hormones are shown in Figs 2 and 3. The mean daily plasma concentrations of 17β-oestradiol remained relatively constant during the studied period, although with a high variability among groups and among individuals within the same group. The comparison showed that both mean daily concentrations (Fig 2A) and area under the curve during the first 60 days of gestation (Fig 2B) remained lower in the sheep maintained at high-altitude, with significant effects of the altitudinal origin of the animals (P<0.01), and also an effect of the altitude where pregnancy was observed (P<0.001). Lower oestradiol concentrations in the group LH (sheep naïve to high-altitude) were found when compared to those of the group HH (sheep native of high-altitude).

The assessment of the mean daily plasma concentrations of progesterone showed, overall, a steady increase with time until Day 13 after mating and therefore a slower increase up to Day 60 of gestation, when the study finalized (Fig 3A). However, the group LH was an exception to this general pattern since the ewes in this group evidenced a decline in mean plasma progesterone concentrations from Day 9 to 17 of pregnancy and a subsequent rise from Day 17 onwards; hence, plasma progesterone concentrations were significantly lower in this LH group than in the other groups at Days 17 and 21 of gestation (P<0.05), but similar from Day 25 of gestation, onwards. The effect of the short-term exposure to high-altitude on plasma progesterone was also reflected in the area under the curve during the first 60 days of gestation (Fig 3B), with the group LH showing significantly lower values than the other groups. The administration of antioxidant vitamins avoided both the decrease in plasma concentrations between Days 9 and 20 and the lower mean concentrations during the first 60 days of gestation. The effect of antioxidant treatment was determined by the interaction between altitude of origin of the animals and the altitude where they developed their gestation (P<0.05); thus, no effect of the vitamins was detected in the other groups.

### Effects of altitude, altitudinal status and antioxidant treatment on embryo-foetal growth

The first visualization of the embryonic vesicles was possible at day 15.9 ± 3.4 of pregnancy, in average, with a trend for a later visualization in the sheep allocated at high-altitude (17.1 ± 2.8 vs. 13.0 ± 3.1 days for high- and low-altitude sheep, respectively; P = 0.06). No significant difference between groups HH and LH was found. The first visualization of the embryo heartbeat occurred at an average of 22.3 ± 3.2 days of gestation, without significant differences among groups.

**Fig 4:**
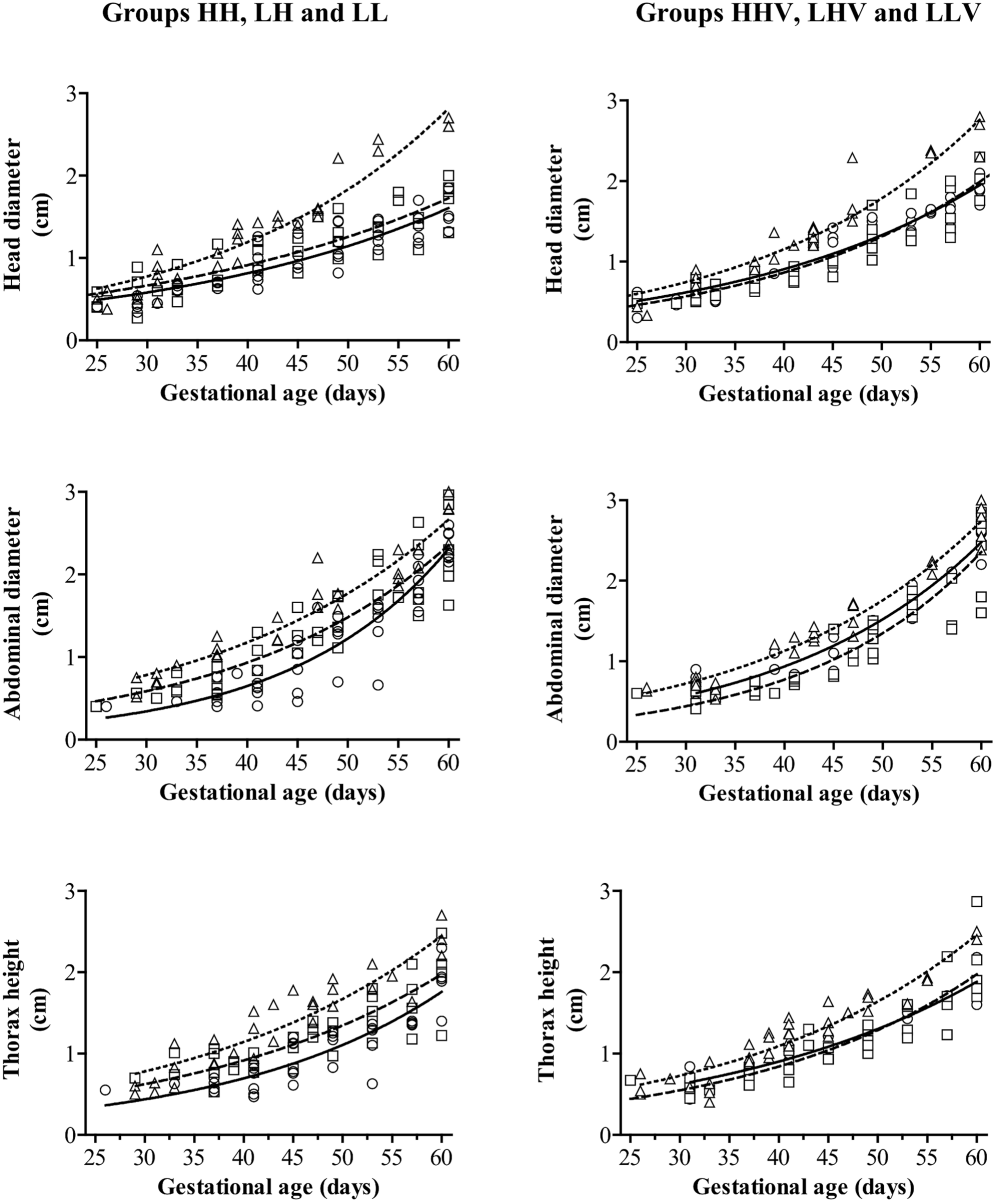
Growth patterns of head, thorax and abdominal diameters in lamb fetuses from mothers maintained at high or low altitude during pregnancy. Panels at left hand represent biometric measurements from foetuses in the groups with different altitudinal status and without supplementation with antioxidant vitamins (n = 6 each); HH: ewes native to highlands whose gestation took place at high altitude (circles and solid lines); LH: ewes native to lowland whose gestation took place at high altitude (squares and dashed lines); LL: ewes native to low-altitude maintained at low-altitude (triangles and dotted lines). Panels at right hand represent biometric measurements from foetuses in the same altitudinal status groups but supplemented with antioxidant vitamins (HHV, LHV and LLV); symbols and lines are the same as in the left panel. The growth-slopes of head, thorax and abdomen were lower at high-altitude (solid and dashed lines) than at the sea-level (dotted lines; P<0.001 for head and P<0.05 for thorax and abdomen). Foetuses LH showed a higher thorax growth-slope than the HH (P<0.01). No significant effects of vitamin supply on the growth curves of head and thorax was obtained for three groups (P>0.05). However, the administration of vitamins showed a significant effect increasing the abdomen diameter in the groups HHV and LHV (P<0.01 for both).

The assessment of the embryo-fetal developmental trajectory showed that the embryo-fetal size was affected by exposure to high-altitude from the initial measurements at Day 25 of pregnancy and throughout the first 60 days of gestation (Fig 4). The changes over time in embryo/fetal head (Fig 4A), thorax (Fig 4B) and abdominal diameters (Fig 4C) were best adjusted to exponential growth in all the groups (r^2^>0.97 for all of them). The growth-slopes of the head, thorax and abdomen were lower at high-altitude (groups HH and LH) than at those of the sheep fetuses at the sea-level (group LL; P<0.001 for head and P<0.05 for thorax and abdomen, when compared to both HH and LH). There were no significant differences in the values of the head and abdomen development at high-altitude, but the group LH showed a higher thorax growth-slope than the group HH (P<0.01).

There were no significant effects of the administration of antioxidants on the timing for first visualization of the embryonic vesicle or embryo. There were also no effects of the vitamin supply either on the growth curves of head and thorax diameters among any of the three groups (P>0.05), although head diameter was larger in the group HHV when compared to groups LH and LHV (P<0.005 for both). Conversely, the administration of vitamins showed a significant effect increasing the abdomen in the groups HHV and LHV (P<0.01 for both), with this trait in the group HHV showing a similar slope than in groups LL and LLV (P = 0.15). Finally, at birth, bodyweight of the lambs was significantly affected by altitudinal status and vitamin treatment (Table 2).

**Table 2:** Body weight (mean±SD) of newborn lambs: effects of long or short time maternal exposure to altitude and of maternal supplementation with vitamins C and E.

	LL	LLV	LH	LHV	HH	HHV
Birthweight (g)	4420±379^a^	4332±387^a^	2732±650^d^	3559±319^bc^	3189±397^b,c^	3748±258^b^

Different superscript letters indicate significant differences among groups (P<0.05). LL: newborn lambs from low altitude native mothers maintained at low altitude, without vitamins supplementation; LLV: newborn lambs from low altitude native mothers maintained at low altitude, supplemented with vitamins; LH: newborn lambs from low altitude native mothers taken to high altitude, without vitamins supplementation; LHV: newborn lambs from low altitude native mothers taken to high altitude, supplemented with vitamins; HH: newborn lambs from high altitude native mothers maintained at high altitude, without vitamins supplementation; HHV: newborn lambs from high altitude native mothers maintained at high altitude, supplemented with vitamins.

## Discussion

The results of the present study indicate that both long- and short-term exposures to the high altitude hypoxemic environment cause disturbances in maternal ovarian steroidogenic function and negatively affect embryo-fetal growth from conception and early pregnancy stages. The discrete effects of the supply of antioxidant vitamins suggest only a limited involvement of oxidative stress in the occurrence of IUGR at the beginning of pregnancy at high-altitude.

Actually, although it was not an objective of the study, both long- and short-term exposures to the high altitude hypoxemic environment may have caused disturbances in the reproductive function during the periconceptional period. The 36 animals that were selected for the study arose from a group of 60 females that were synchronized and inseminated (20 LL, 20 LH and 20 HH). The fertility rate was different among groups (100% for LL and LLV, 70% for LH and 80% for LHV, and 80% for HH and HHV), with a significant difference among sheep exposed to altitude and sheep maintained at lowlands (P<0.001). Administration of vitamins showed no effects on fertility of sheep in groups LLV and HHV when compared with their counterparts, but increased fertility in LHV group (P<0.05 when compared to LH).

The assessment of markers for blood oxygen transport in the sheep exposed to high-altitude in the current study evidenced hypoxemia in all of them, native and naïve to altitude sheep (groups HH and LH, respectively), and also indicated a beneficial effect of the antioxidant vitamins therapy, in agreement with previous studies of our group [31, 41, 42].

The state of hypoxemia in sheep exposed to high-altitude was associated with deficiencies in steroidogenic function and with stronger disturbing effects in the newcomer sheep. Low plasma oestradiol concentrations in both groups HH and LH, were found, with the group LH being the one with lowest values. To our knowledge, there are scarce studies on the effects of hypobaric hypoxia and/or hypoxia-induced oxidative stress on estrogen secretion during pregnancy of animals and/or humans, and most of them are referred to late pregnancy. However, the results of the present study are in agreement with data obtained at later pregnancy stages in our sheep model (from Day 60 of pregnancy onwards; [31]). In humans, women developing gestation at high-altitude evidenced lower plasma oestradiol concentrations than those concentrations in low-altitude pregnant women at 30 weeks of gestation and onwards [43]. These effects may be explained by a decrease in aromatase activity when the partial pressure of oxygen is decreased, as described for trophoblast cells in culture [44]. Deficiencies in oestradiol secretion may be a presumptive causal factor for IUGR at high-altitude, since low plasma estradiol concentrations have been previously found in pregnancies with fetal restriction at low-altitude [45]. Concurrently, circulating oestradiol levels have been recently associated with an adequate development of uterine artery diameters and blood flow at high-altitude [46] and it is well-known that adequate placental growth, placental development rate and placental vascularization favor fetal development, fetal size and weight of the neonates [47].

The pattern of plasma progesterone concentrations was similar in sheep native to and maintained at both, high- and low-altitude (groups HH and LL), since the trend of the group HH to show higher values than the other groups did not reach statistical significance. On the other hand, the group LH showed similar values to HH and LL until Day 9 of pregnancy, when a sharp decrease with time in progesterone concentration between days 9 and 17 of pregnancy was found; since Day 25 of pregnancy progesterone showed again similar values to those of the other groups. Previous studies performed in the same conditions with cyclic, non-pregnant sheep [42] showed similar results at the early-luteal phase but did not evidence any decrease in plasma progesterone concentrations during the late luteal-phase; on the contrary, the values of progesterone in blood were significantly higher in sheep from both groups HH and LH, than in the sheep of group LL. The recovery of plasma progesterone values after Day 25 may be related to the beginning of the placental secretion of progesterone. The knowledge about the placental progesterone secretion function during the first two months of pregnancy in sheep is limited, but studies starting at 35 and 40 days of pregnancy [48, 49] have found evidence of progesterone secretion by the placentomes.

In previous studies of our group, we observed even higher plasma progesterone concentrations at the end of the pregnancy in LH sheep when compared to HH sheep [31] although there were no LL counterparts in that study. To the best of our knowledge, there is no other related information in the literature, so these discrepancies open a target for more specific researches. In any case, these data seem to support our previous hypothesis addressing that the variations found in plasma progesterone concentrations in sheep exposed to high-altitude may be more related to differences in progesterone metabolism rather than in progesterone secretion itself, since plasma progesterone levels are known to be more affected by metabolic clearance than by the level of secretion [50].

The first finding about the effects of altitude during the very early-pregnancy in the current study consists on a later detection of the gestational vesicle. At low-altitude, the gestational sac was detected around Day 13 after mating, concomitantly with the dates usually described when using a high resolution ultrasound probe, independently of the experience of the sonographer (Day 12; [51, 52]). Conversely, the gestational sac was observed four days later in the animals maintained at high-altitude and it is logical to assume that a smaller size of the embryonic structure influenced its ultrasonographic visualization. On the other hand, the lack of differences in the day of first embryo visualization is very likely caused by the fact that the data were based on heartbeat detection, which is more determined by the detection of a pulsating structure inside the embryo than by the size of the proper embryo. Nevertheless, the embryos/fetuses from high-altitude pregnancies were significantly smaller than conceptuses from low-altitude pregnancies, with scarce differences between sheep native and naïve to high-altitude, from the first measurements at Day 25 of pregnancy and throughout the first 60 days of gestation.

Thus, our results indicate that IUGR under hypobaric hypoxia may take place not only at advanced gestation stages, but from the earliest stages of pregnancy, and, hence, that a short-term exposure to altitude during the early-pregnancy may affect conceptus development. These findings might be related to a lower uterine oxygenation reducing oxygen supply to the conceptus after placentation. However, the hypoxic state of the uterine environment during pre- and peri-implantational stages is well-characterized [20–22]; consequently, the very early-pregnancy IUGR may be caused by indirect effects of the superimposed maternal hypoxia. In very early embryos, growth and development rates depend largely on a proper IGF-axis balance, which components are expressed by the embryo, the oviduct and the uterus [53, 54]. The early embryonic development is highly dependent on the histotroph [55] and on the uterine fluid, which is rich in proteins, enzymes, carbohydrates, hormones, ions and growth factors, including IGFs [56]. We have recently demonstrated that, in the luteal tissue, exposition to a hypoxic environment reduces the IGF-I and II expression [42], so we can assume a similar effect on the expression of these growth factors in the uterine and embryo tissues resulting in a reduction of embryo growth. However, based on the current findings, more specific studies need to be developed.

Alternatively, the effects of hypobaric hypoxia during the early-pregnancy may be related to hypoxia-related oxidative stress since nutrition of the embryos, before completion of the placenta, is achieved by phagocytic activity of the trophoblast [57, 58], activity which is mediated by reactive oxygen species [59]; hence, increased oxidative stress may affect embryo development [23]. However, the supply of the same antioxidant treatment being successful for increasing birth-weight at late-pregnancy stages [31] showed little effect on the early-pregnancy, so the evidence points to a limited involvement of oxidative stress in the occurrence of IUGR at the beginning of pregnancy at high-altitude. However, additional studies on the effects of antioxidant treatments may need to be performed having in mind the results regarding birth-weight and viability of the neonates in the current and previous studies [31]. Moreover, when there is strong recent evidence of positive effects of antioxidant treatments in the asymmetry of growth pattern and blood flow redistribution which are main characteristics of IUGR in case of hypoxia [60–63].

In conclusion, the present study indicates that the exposure to high-altitude during the very early stages of pregnancy, even in a short-term, affects the maternal ovarian steroidogenic function and the embryo-fetal growth. Thus, our results give a warning for clinicians about the risks for early-pregnant women when visiting high-altitude regions and give way to further studies on the effects and interactions of the length of the exposure to altitude with the pregnancy stage.
